# Understanding the nature and mechanism of foot pain

**DOI:** 10.1186/1757-1146-2-1

**Published:** 2009-01-14

**Authors:** Fiona Hawke, Joshua Burns

**Affiliations:** 1 Podiatry Department, School of Health Sciences, Faculty of Health, University of Newcastle, NSW, Australia; 2 Institute for Neuroscience and Muscular Research, The Children's Hospital at Westmead/Discipline of Paediatrics and Child Health, Faculty of Medicine, The University of Sydney, NSW, Australia

## Abstract

Approximately one-quarter of the population are affected by foot pain at any given time. It is often disabling and can impair mood, behaviour, self-care ability and overall quality of life. Currently, the nature and mechanism underlying many types of foot pain is not clearly understood. Here we comprehensively review the literature on foot pain, with specific reference to its definition, prevalence, aetiology and predictors, classification, measurement and impact. We also discuss the complexities of foot pain as a sensory, emotional and psychosocial experience in the context of clinical practice, therapeutic trials and the placebo effect. A deeper understanding of foot pain is needed to identify causal pathways, classify diagnoses, quantify severity, evaluate long term implications and better target clinical intervention.

## Background

Foot pain is experienced by 17 to 42% of the adult population [1-4]. It is disabling in nearly half of these cases [4] and can impair mood, behaviour, risk of falls, self-care ability and quality of life [3,5-11]. Foot pain is complex, and difficulties in accurately diagnosing the source of pain and cause of tissue damage can impair clinical management of the pain [12,13]. However, most people with foot pain do not seek professional treatment, even when the pain is disabling [4]. There is clearly a need to improve the provision of foot care to people suffering such pain.

Currently, the aetiological mechanisms underlying some types of tissue injury within the foot are not clearly understood. As a result, interventions targeting foot pain in clinical trials often lack specific targets (*e.g. *plantar heel pain) [14]. Perhaps as a result of this limitation, evidence from randomised controlled trials of some common interventions that are highly regarded in clinical practice (*e.g. *custom foot orthoses) have detected only small, if any, beneficial effects [15].

A deeper understanding of pain is needed to identify the nature and mechanism of foot pain, its diagnosis and how best to target clinical intervention. It has been two decades since a review on foot pain has been published [16-19]. Given that almost all prevalence studies for foot pain have been performed since then, in addition to the recent advances in our understanding of the nature and mechanism of pain in general, a review of this type is warranted. The aim of this paper was to comprehensively review the literature on foot pain, with specific reference to its definition, prevalence, aetiology and predictors, classification, measurement and impact. We conclude by discussing the complexities of foot pain as a sensory, emotional and psychosocial experience in the context of clinical practice, therapeutic trials and the placebo effect.

## Defining foot pain

Foot pain is an unpleasant sensory and emotional experience following perceived damage to any tissue distal to the tibia or fibula; including bones, joints, ligaments, muscles, tendons, apophyses, retinacula, fascia, bursae, nerves, skin, nails and vascular structures [20]. Foot pain is a general term, inferring neither pain class, injury mechanism nor histological pathology. As further discussed in later sections, it is important to recognise that foot pain is not the noxious-stimuli-induced activity in the nociceptive pathways [20,21], but rather the perception of these processes and the consequent effects on suffering and pain-related behaviour [22].

## Prevalence of foot pain

Few studies have investigated the prevalence of foot pain in large, randomly selected samples. Instead, attention is typically given to specific pathology (*e.g. *heel pain) or population groups (*e.g. *people over 65 years of age). A summary of studies reporting the prevalence of general foot pain in randomly selected samples is presented in Table 1. Overall, it is thought that foot pain affects 14 to 42% of people at any given time depending on definition and measurement of pain, sample characteristics (age, gender) and study location. Garrow *et al*. [4] found that the most commonly reported foot pain sites among people reporting symptoms of disabling foot pain (defined in Table 1) were the mid-foot/arch area (25.6%), first metatarsal head (20.2%), great toe (15.9%) and plantar surface of the heel (15.5%). Further research is required to characterise the exact types of foot pain in the general community.

**Table 1:** Prevalence of foot pain in randomly selected populations

**Study**	**Sample source and description**	**Foot pain prevalence**	**Pain outcome measure and notes**
Hill 2008	4,060 people aged ≥20 yrs (51% female) recruited by telephone interview (49% response rate) from north-western Adelaide, South Australia	17%	Foot pain defined as affirmative response to 'On most days do you have pain, aching or stiffness in either of your feet?'
Menz 2006	301 community-dwelling older adults (representing 31% response rate) aged 70–95 yrs (61% female) from Sydney, NSW, Australia	36% disabling	Disabling foot pain defined as: current foot pain, foot pain in the past month, plus at least one item marked on the Manchester Foot Pain and Disability Index [4].
Badlissi 2005	784 community-dwelling older adults (representing 85% response rate) aged 65–101 yrs (57% female) from Springfield, Massachusetts, USA	42%	Foot pain defined as: at least 'fairly often' foot pain in the previous week, or foot pain or discomfort 'most days' within the previous month [1].
Garrow 2004	3,417 community-dwelling adults (representing 84% response rate) aged 18–80 yrs (55% female) from North Cheshire and Manchester, England:	22% (9.5% disabling)	Foot pain defined as: foot pain during the past month lasting at least one day. 'Disabling' foot pain defined using the Manchester Foot Pain and Disability Index (defined above) [5].
Menz 2001	135 community-dwelling older adults, all members of one private health insurance company (response rate of 28%)aged 75–93 yrs (59% female) Sydney, NSW, Australia.	21%	Foot pain defined as: affirmative answer when asked whether they suffered from painful feet [7].
Leveille 1998	990 community-dwelling women (70% response rate) with a disability; aged 65 to ≥85 yrs from Baltimore, Maryland, USA	18% moderate (14% chronic and severe)	Chronic and severe foot pain defined as: 7–10 on 10-point VAS for ≥1 month within the last year and present in the previous month. Moderate foot pain defined as: 4–6 on VAS for ≥1 month within the last year, or pain rated as 7–10 on VAS lasting ≥1 month and not present within the previous month [10].

## Aetiology of foot pain

Tissue damage in the foot may occur via chemical, mechanical or thermal stimulation [23] associated with direct trauma, musculoskeletal overload, infection, or systemic or proximal pathology (*e.g*. nerve entrapment, diabetic neuropathy). Many common types of foot pain such as tendonitis, stress fracture, corns and callus are routinely attributed, in part or full, to mechanical stress [24]. While mechanical stress (broadly defined as force applied to tissue) is a normal component of foot function, tissue damage occurs when the maximum stress threshold of the tissue is exceeded [25]. This may occur with: (1) short duration, high magnitude stress; (2) long duration, low magnitude stress; or (3) repetitive moderate-magnitude stress [26].

### Associations and predictors of foot pain

Identifying factors that predict foot pain enables the clinician to modify or prevent contributing factors and even target at-risk groups with preventative strategies and more appropriate treatments. Demographically, advancing age and female gender are associated with foot pain [4]. However, while the prevalence of disabling foot pain has been shown to increase with age in both genders peaking at 55 to 64 years of age (15% for females and 12% for males), it has been reported to then steadily reduce with older age [4]. In contrast, studies specifically focussing on foot pain in older adults suggest otherwise, with prevalence as high as 42% (Table 1).

Disabling foot pain appears to occur typically in association with other pain regions, including hip/leg pain, axial skeletal pain and/or shoulder pain; and is more likely to occur in patients previously diagnosed with arthritides, diabetes and/or stroke [4,5,10]. In the largest study to date, Garrow *et al. *[4] reported people with rheumatoid arthritis were three times more likely to report disabling foot pain, although this did not reach statistical significance due to the very small number of people included in this part of the analysis.

Garrow *et al. *[4] also reported that people in Northwest England aged 18 to 80 years with disabling foot pain were significantly more likely than people without disabling foot pain to self-diagnose nail problems (42% vs. 22%), corns and callosities (41% vs. 30%), bunions (19.5% vs. 7%), swollen feet (34% vs. 10%), flat/planus feet (9% vs. 6%), high arch/cavus feet (18% vs. 13%) and toe deformity (33% vs. 13%) (*p *< 0.05). Menz *et al. *[5] also reported associations between disabling foot pain and pes planus as well as limited ankle joint range of motion in older Australians. In the study be Garrow et al [4], however, podiatrist-diagnosed foot problems using established criteria [27-29] revealed only swollen feet as a correlate of disabling foot pain (43.7% vs 18.0%; OR: 3.8; 95% CI: 1.7 to 8.2). This unexpected result is supported by Badlissi *et al. *[1], who reported that people over 65 years of age with foot pain were no more likely than people without foot pain to have hallux valgus, pes planus or lesser toe deformity (including hammer, mallet, claw or overlapping toes and bunionette). Badlissi [1] did note, however, an association between foot pain and pes cavus. Discrepancies between these studies are possibly due to differences in sample characteristics and diagnostic/classification criteria.

Extrinsic factors commonly associated with foot pain include inappropriate footwear [30,31] and occupational activities [32], although these areas have received little empirical investigation in the past. For both intrinsic and extrinsic factors, further research is needed to develop predictive models of foot pain causation in large prospective random samples of children, adolescents and adults.

## Classification of foot pain

The difficulties in clearly defining pain have impeded the development of clinically relevant pain models capable of guiding foot pain classification and communication among and between practitioners and patients [33-35]. Currently, emerging evidence of the neurological differences between physiological and pathophysiological pain is prompting the redevelopment of existing pain classification models, particularly for chronic pain, which will have implications on our understanding of foot pain [36-38]. The following section clarifies the underlying neurological differences between the many clinical presentations of foot pain, although it is important to point out that many aspects of foot pain are not mutually exclusive.

### Physiological foot pain

Physiological foot pain is experienced as an acute response to injury (or potential injury) following healthy functioning of both the peripheral and central nervous systems [37,39]. It provides a feedback system to encourage the removal of potential tissue-damaging stimuli (as per defense-response theory) [35,37,40]. There are three essential criteria for classification as physiological foot pain [23,35,37-39]: (1) noxious (potentially tissue damaging) stimuli are extrinsic to the nervous system; (2) pain perception is proportionate to the magnitude of noxious stimulation; (3) pain diminishes when the stimuli are removed. An example of physiological foot pain would be the response to a stone trapped in one's shoe or a blister from a new pair of shoes. The activity within the nervous system producing the experience of pain is termed nociception. Nociception in physiological foot pain comprises three distinct processes: transduction; transmission; and modulation.

#### Transduction

Foot pain is the end result of a cascade of impulses originating in the stimulation of structurally unspecialised free nerve endings within foot tissue [23,41]. These free nerve endings are called nociceptors. In response to potentially harmful mechanical, thermal and chemical stimuli, nociceptor cell membranes depolarise. If the stimulation is strong enough, ion channels within the membrane are activated; creating a self-propagating change in membrane potential that sweeps along the electrically excitable membrane cells [23,38].

#### Transmission

Nociceptors within the feet are capable of both efferent and afferent transmission [35]. Efferent transmission of the action potential (back to the site of stimulation) causes the release of neurotransmitters and neuropeptides from peripheral fibre terminals, producing the classic 'axon reflex': neurogenic inflammation at the site of tissue damage [23,37]. Afferent transmission (away from the foot) occurs *via *two types of primary afferent nociceptive neurons: A-delta fibres and C fibres [22,36]. The roles of these fibres in nociception from the foot are outlined briefly in Table 2[23,35,38]. A-delta fibres and C fibres of the primary afferent nociceptive neurons travel from the foot to synapse with second-order neurons in the superficial layers of the spinal dorsal horn [22,23]. Second order neurons contralaterally ascend the spinal cord *via *several pathways [37], of which the spinothalamic pathway is regarded as the most important for nociception [38]. At this level, second order neurons activate lower motor neurons in the spinal ventral horn, provoking a reflex withdrawal from the noxious stimulus (*e.g. *jerking the foot away from splintered wood) [23]. Clinically, disruption of this protective reflex can be observed in some sensory and lower motor neuropathies including Diabetes Mellitus and Charcot-Marie-Tooth disease. Second order neurons ascending the spinothalamic pathway synapse with third order neurons in the thalamus. From the thalamus, impulses are propagated to the primary somatosensory cortex, where the discriminative components of pain are perceived, and to limbic cortical areas, where the affective and emotional aspects of the pain experience are perceived [23,35,38]. While these pathways are complex, it is important to maintain a clinical appreciation of the various levels at which dysfunction can occur and therapy can target.

**Table 2:** Roles of A-delta and C fibres in nociception

**Role**	**A-delta fibres**	**C fibres**
**Myelination**	Thinly myelinated	Unmyelinated
**Neuronal diameter**	1 to 5 microns	< 1.5 microns
**Conduction speed**	5–20 metres per second	0.5–2 metres per second
**Stimuli**	Mechanical and sometimes thermal	High intensity mechanical, thermal and chemical
**Pain sensation**	Fast	Dull, throbbing, aching

#### Modulation

Mechanisms capable of modifying the propagation of nociceptive impulses from the foot to the brain have been proposed to exist at all levels of the nervous system and to influence both sensory and emotional components of pain [35,38,42]. This selective projection and inhibition of impulses has been attributed in part to neural plasticity (the ability of neural tissue to regulate its own activity) [35]. The foundations of neural plasticity were first introduced in the Melzack-Wall gate control theory of pain in 1965 [43]. Melzack and Wall hypothesised that afferent impulses (ascending toward the brain) could be inhibited by efferent impulses (descending from the brain) in the dorsal spinal horn. Recent research has supported Melzack and Wall's hypothesis and highlighted the influence of psychosocial factors (*e.g. *pain beliefs) on the descending inhibition and consequent reduced experience of pain [22,23,36]. Modulation of nociception might account for some of the foot pain reduction experienced with the placebo effect.

### Pathological foot pain

Pathological foot pain is experienced following nociceptive pathology; involving dysfunction of either or both of the peripheral or central nervous systems [37,39]. While there is debate as to which classes of pain deserve categorisation as pathological foot pain, common suggestions include neuropathic, inflammatory and chronic pain [36,37]. These pain classes are categorised as pathological foot pain since at least one of the three criteria for physiological foot pain is not met [23,35,37-39]. That is, in pathological foot pain: (1) noxious stimuli are intrinsic to the nervous system; (2) foot pain perception is disproportionate to the magnitude of noxious stimulation; and/or (3) foot pain does not diminish when the stimuli are removed. Due to such dysfunction, pathological foot pain extends far beyond the mechanistic defense response role attributed to physiological foot pain [37].

#### Neuropathic foot pain

Neuropathic foot pain is pain instigated by a primary dysfunction, lesion or transitory perturbation in the peripheral or central nervous systems [20]. Neuropathic foot pain encompasses a heterogenous group of symptoms that share similar clinical characteristics, including spontaneous stimulus-dependent and stimulus-independent pain. Spontaneous foot pain typically appears incompatible with the initial cause and affected anatomical site, and often has unpredictable treatment responses [39,44-46]. A summary of the characteristics of neuropathic foot pain is presented in Table 3[20,44], however the mechanisms underlying these clinical characteristics are not fully understood [39]. Symptoms have been proposed to reflect reactive hyperexcitability and sensitisation of peripheral and central neural elements, and relative suppression of central inhibitory pathways following central nervous system damage [39,44,47]. Changes include abnormal ion channel expression due to disruption of normal neuronal input and pathological activation of injured nerve fibres by inflammatory mediators and sympathetic excitation [44,48]. These changes reduce depolarisation threshold, resulting in spontaneous, ectopic discharges [41]. The ensuing hectic and persistent neural activity can cause ephaptic conductions (electrical connections between injured and adjacent uninjured nerve fibres) [39]. The anatomical site of these changes may be at any level within the nervous system, from peripheral receptor within the foot to the highest cortical centres [44]. Ephaptic conductions might account for some clinically confusing presentations of foot pain and might underlie the spreading of pain experienced by some people. It is not clear from the literature whether ephaptic conductions form between afferent (sensory) and efferent (motor) fibres. If interfibre-type connections do occur, these might account for some motor disturbance in cases of neuropathic pain, *e.g. *autonomic dysfunction in complex regional pain syndrome type I [49,50].

**Table 3:** Clinical characteristics of neuropathic foot pain [20,44]

**Characteristic**	**Definition**
**Allodynia**	Evocation of pain by a stimulus that does not normally evoke pain.
**Dysthesia**	A spontaneous or evoked unpleasant, abnormal sensation, *e.g. *hyperalgesia and allodynia.
**Hyperalgesia**	Increased pain response to a stimulus that is normally painful. Might be static, punctate or dynamic, and might occur with thermal stimuli. Suggested to be a consequence of peripheral and/or central sensitisation.
**Hyperesthesia**	Increased sensitivity to stimulation, including diminished threshold and increased response. Excludes the special senses.
**Hyperpathia**	Increased threshold and abnormally painful reactions to stimuli, especially repetitive stimuli. Might occur with dysthesia, hyperalgesia, allodynia or hyperesthesia. Occurs in the presence of fibre loss.
**Paraesthesia**	A spontaneous or evoked, abnormal but not unpleasant sensation. Proposed to reflect spontaneous bursts of A-β fibre activity.
**Paroxysms**	Spontaneous or stimuli-associated shooting, electric-shock like or stabbing pains. Might be elicited by an innocuous tactile stimulus or by a blunt pressure.
**Referred pain**	Abnormal spread of pain from a peripheral or central lesion. Typically referred from deep to cutaneous structures.
**Sensory deficit**	Partial or complete loss of afferent sensory function. Might not involve all sensory pathways.

Neuropathic pain is routinely sub-categorised according to the causative factor, *e.g. *mechanical injury, neurotropic viral disease, neurotoxicity, metabolic disease, inflammatory and/or immunologic mechanisms, focal ischaemia or neurotransmitter dysfunction [47]. It is expected that continued advances in molecular neurobiology will expose links between sub-categories and allow for the development of a comprehensive and coherent classification system for neuropathic foot pain [39,44].

#### Inflammatory foot pain

'Inflammation' describes a wide range of primarily vascular responses to tissue injury [51]. Pain (*dolor*) is one of the five classic, clinical features of acute inflammation, along with redness (*rubor*), heat (*calor*), swelling (*tumor*) and limitation of function (*functio laesa*) [52]. Inflammation produces characteristic changes within the nervous system [53]. In early stages, inflammatory mediators activate second-messenger systems, thereby sensitising polymodal nociceptors and reducing the activation thresholds of conducting ion channels [36,41,54]. Within the foot, cutaneous nociceptors are sensitised to thermal stimuli and deep somatic nociceptors are sensitised to mechanical stimuli [41]. Clinically, this can be observed as abnormally painful responses to surface temperature changes (*e.g. *application of ice) and/or palpation and physical movement of affected joints. During this process, 'silent' or 'sleeping' nociceptors within the foot may be activated [36,37,55]. Once activated, these nociceptors fire persistently to produce uninterrupted pain [23]. Longer term, cytokine and growth factor mediated transcription is accelerated, increasing the rate of receptor production [22]. As a result, primary hyperalgesia occurs at the site of tissue damage [36]. These changes are frequently accompanied by sensitisation of the central nervous system and nerve damage, which may provoke neuropathic foot pain [36].

#### Chronic foot pain

Proposed definitions of chronic pain are inconsistent and difficult to use in clinical practice [34,37]. Despite its widespread use, the term 'chronic' has been criticised for its potential to be confusingly used as a descriptor of pain history and as a prognostic statement for pain [34]. The International Association for the Study of Pain (IASP) defines chronic pain as any pain persisting past the normal time of healing and suggests three months to be the most suitable point of division between acute and chronic pain for nonmalignant pain [20]. Variations to this definition are common, particularly with regards to time framing [20,37,56].

Despite semantic disagreement, there is apparent consensus regarding clinical and underlying physiological distinctions between acute and chronic pain [21]. Chronic foot pain does not typically share the sharp spatial localisation typical of acute foot pain. Chronic foot pain is characteristically diffuse, spreads beyond the original site of injury, exhibits a non-linear relationship between nociception and pain intensity, and involves adaptive changes at various levels of the nervous system, *e.g. *activation of propriospinal reflexes, which play a role in coordination, posture and locomotion [21,35,41].

Clinically, it is important to recognise that chronic foot pain is pain persisting past the normal time of healing following the removal of the noxious stimulus [20]. Chronic foot pain is not simply pain persisting past an arbitrary time point (*e.g. *three months). If the stimulus has not been removed, the pain should not be termed chronic.

### Changes in foot pain perception with age

In recent years, several comprehensive reviews have discussed age-related changes in pain perception [57,58]. Whilst there is some contradiction between empirical findings, most studies demonstrate age-related increases in pain threshold (the least amount of stimulation required for a person to experience pain) using heat or mechanical stimulation, but not from electrical stimulation [59]. The decline in heat pain sensitivity is most noticeable after 70 years of age and may be more pronounced in the distal extremities [60]. Pressure pain threshold increases by about 15% and is more noticeable in females than males [61]. Heat pain threshold increases by about 20% for radiant pain and 50% to 100% for CO_2 _laser pain [59,62].

Whilst there appears to be a modest age-related increase in pain threshold and diminished sensitivity to low levels of noxious stimulation, response to higher intensity stimuli is increased and tolerance of strong pain is reduced [59]. Recent experimental studies suggest this may stem from alterations in peripheral A delta and C fibre nociception and central nervous system changes, including reduced central nervous system plasticity following injury and reduced efficacy of endogenous analgesic mechanisms [59].

## Quantifying foot pain

There is currently no universally accepted standard for the measurement of pain [63]. As a result, numerous quantitative and qualitative pain measurement tools have been developed. Since pain is a subjective sensory and emotional experience, the participant's own reporting of pain is widely regarded as the most valid representation of their pain [63]. As such, self-reported pain intensity is the most frequently used research tool to measure pain [44,64]. Popular tools include visual analogue scales (VAS), numerical rating scales and verbal category/Likert scales [44,64,65]. Tools used to measure foot pain include the: Foot Function Index [66]; Foot Health Status Questionnaire [67], physical health domains of the Diabetes Foot Ulcer Scale [68]; Manchester Foot Pain and Disability Index [69]; Rowan Foot Pain Assessment Questionnaire [70]; American Academy of Orthopaedic Surgeons Foot and Ankle Questionnaire [71]. Across all these tools, the individual's subjective reporting of pain is regarded as a valid representation of *their *pain [63]. However, criticism of pain intensity outcome measures have concluded that: people preferentially use the beginning, middle and end of continuous pain scales (*e.g. *VAS) [63]; there are specific clinical attributes of pain class not always captured in generic tools (*e.g. *chronic/inflammatory/neuropathic) [44]; the fluctuating nature of many pain conditions are often inappropriately disregarded [44]; the results of intervention trials are often difficult to interpret due to unknown or unspecified clinically important differences detected by the pain measurement tool used [63].

Despite these limitations, foot pain as an outcome measure has much to offer clinical practice and research [65]. It is important, however, to ensure that pain reduction does not dominate health outcome assessment in clinical practice. Jensen *et al. *[44] suggest that pain reduction has dangerously been equated with therapeutic success, leaving many other clinically relevant health outcomes overlooked, *e.g. *functional ability.

## Impact of foot pain

Considering the combined sensory and emotional components of pain, pain has the potential to produce effects far surpassing the auto-protective role depicted by the defense response mechanism [9,64,72-93]. A summary of the impacts of pain in general is presented in Table 4. Foot pain specifically has been associated with reduced functional ability, including self-care [3,8-11], increased risk of falls [6], depression [5] and reduced physical and mental aspects of quality of life [94]. While these effects are much less extensive than those associated with pain in general (Table 4), relatively few studies have evaluated the impact of foot pain and the outcomes assessed have been limited in scope.

**Table 4:** The broader impact of pain in the community

**Domain**	**Impact**
**Social life**	Inability to pursue hobbies among children and adolescents [72] Reduces social functioning among children, their families and older adults [73-75] School absenteeism among children and adolescents [72]
**Physical function**	Fear of movement and re-injury in chronic musculoskeletal pain [76] Reduced physical functioning among children, adolescents, adults and older people [9,64,77-81]
**Mental function**	Sleep disturbances among children, adolescents and older people [72-74,77] Mood disturbances among adolescents and older people [73,77] Interpersonal strain due to behavioural changes among children and their families [75] Increases depressive symptoms, particularly if accompanied by self-blame [82-84] Increases severity of depressive symptoms [85]
**Overall impact**	Reduces quality of life [73,74,77,83,86,88,89]
**Health care**	Increases prescription/consumption of analgesic drugs [80,87,90] Impairs recognition of depression [91] Impairs adherence to medication if coinciding with depression [93]

To gauge the full impact of foot pain on one's life, it can be useful to measure health-related quality of life. Health-related quality of life is an individual's health status encompassing any aspect of life affected by mental and physical well being [95]. In recent years, health-related quality of life has been increasingly promoted as one of the most important outcomes for the evaluation of therapeutic interventions for pain [88,96,97]. Pain has a detrimental effect on all aspects of health-related quality of life, spanning all age groups, pain types and pain sources [97]. Of clinical importance is that health-related quality of life is reduced most when pain is of long duration and high intensity [98]. From a study of 81 chronic pain sufferers, Dysvik *et al. *[88] identified five predictors of poor health-related quality of life in chronic pain sufferers: (1) female gender; (2) longer pain duration; (3) greater pain intensity; (4) a view of pain as mysterious; and (5) less social support. Clinically, it might be beneficial to address the modifiable predictors: pain intensity (*e.g. *by therapy); view of pain as mysterious (*e.g. *by education); and less social support (*e.g*. by providing contacts for local support networks).

Some specific tools used to measure health-related quality of life in foot pain research include: four domains of the 36-Item Short-Form Health Survey (physical functioning, general health, vitality, and social functioning) [99]; the Quality of Life subscale of Foot & Ankle Outcome Score [100]; and the Health-Related Quality of Life Index [101]. Evidence from randomised controlled trials demonstrates that effective treatment of foot pain can lead to clinically important improvements in health related quality of life [15].

## Foot pain as a sensory, emotional and psychosocial experience

The biopsychosocial framework depicts foot pain as a result of interaction between biological, psychological and social factors [102]. These include somatic nociceptive input, pain beliefs, coping strategies, mood, social context, cultural context and personal expectations [103,104]. The cognitive behaviour model similarly promotes the influence of psychological and emotional experiences on pain, linking pain beliefs to culturally shared values and powerful emotions [88,105].

While the suggestion that psychological and social factors influence pain experience and treatment outcomes is not new [106], it is only recently that the biopsychosocial and cognitive behavior models have been supported by empirical research. Psychosocial environment and pain beliefs have been shown to affect: how pain is reported [107,108]; the intensity of the pain experienced [84,109]; physiological symptoms [84,109-111]; the development, maintenance and exacerbation of disability [76,88,110]; risk for future musculoskeletal pain [112,113]; and treatment outcomes [84,109]. One important example is the differences in pain experience and report between males and females. Empirical research has demonstrated that a woman's average pain threshold and tolerance is significantly lower than the average man's and that women are more willing to report pain, therefore experiencing pain for less time than males [114,115]. These differences are proposed to stem from both first order, biological sex differences and psychosocial factors including gender-role expectations [115].

Further research is required to understand the many facets of foot pain suffering and to identify or develop interventions effective at modifying the 'foot pain experience'. Clinically, this might be particularly useful for pain unresponsive to routine treatment, (*e.g. *painful diabetic neuropathy, fibromyalgia and complex regional pain syndrome type I) and understanding the complexities of the placebo effect.

### The placebo effect – impact of the psychosocial context on treatment response

It is proposed that the psychosocial context (*e.g. *attitudes and expectations) surrounding an intervention contributes to positive therapeutic outcomes [116-118]. This is called the placebo effect and can occur in both clinical trials and clinical practice [119,120]. In clinical trials, researchers may attempt to isolate the placebo effect from the direct physiological effects of an intervention. This is typically achieved by using a pseudo-intervention devoid of intentional biological activity (*e.g. *sugar pill or detuned ultrasound) [119], which is colloquially known as a placebo. The 'placebo effect' is the change in outcomes observed following administration of the placebo intervention. Due to the biologically inert nature of the placebo intervention, the changes observed are routinely attributed to the psychosocial context surrounding the intervention [116]. The term 'placebo effect', however, is sometimes used misleadingly. The placebo effect encompasses only those changes that occur as a direct result of the administration of intervention. For example, the placebo effect can encompass the Hawthorne effect, where a person modifies their behaviour because they know they are being observed/monitored [121]. The placebo effect does not include changes that would have occurred if the placebo intervention was not given, including the natural progression or spontaneous resolution of symptoms and/or signs [120].

Overall, distinguishing between the changes that occurred due to the administration of the placebo intervention and those changes that would have occurred regardless is difficult, and in some cases impossible. There is, however, widespread historical acceptance of the proposal that the 'placebo effect' is more than a mere measurement artefact or reflection of normal disease progression [122]. Indeed, the placebo effect has been described as the most effective intervention known to science; having been subjected to more clinical trials than any other intervention, usually surpassing expectations of effectiveness, and being effective against an apparently limitless range of conditions [123,124]. It is reported that the magnitude of the placebo effect in double-blinded randomised controlled trials has markedly increased since the mid 1980s [125]; now being capable of reducing symptoms by a mean of 35% [120]. Despite such claims, results of meta-analyses evaluating the existence of a placebo effect are contradictory [122,126]. A Cochrane Collaboration systematic review evaluating the effect of placebo interventions across *any *clinical condition did not detect a statistically significant placebo effect in trials for binary outcomes (where treatment response is measured as one of two possible outcomes, *e.g. *death versus alive) or objective outcomes (where outcomes are measured by an observer, *e.g. *blood pressure) [126]. For self-reported continuous outcomes, however, a moderate placebo effect was detected. This effect was even stronger for self-reported pain outcomes [126].

The placebo effect has been acknowledged in reference to clinical trials of custom-made foot orthoses [127]. As with many physical, mechanical and surgical interventions, however, the development of convincing placebo interventions for custom-made foot orthoses is very difficult, and perhaps impossible. As a result, researchers often employ 'sham' interventions [99,128]. Sham interventions are designed to have minimal mechanical effect but to look and feel like the genuine intervention. Consequently, these sham devices often produce some mechanical effect. Disentangling a true placebo effect from the potential mechanical effect of the sham orthoses and from the influence of changes that would have occurred without intervention (*e.g. *natural disease progression) is complex. Despite such limitations, an investigation attempting to understand the mechanisms by which custom-made foot orthoses reduced cavus foot pain reported that the placebo effect accompanying custom-made foot orthoses as an intervention is strong, and capable of producing clinically meaningful changes in symptoms [127].

Many theories attempting to explain the basis for the placebo effect have been proposed, including: (1) increased use of self-distraction strategies; (2) reduced anxiety (a key emotional component of pain); and (3) expectation of improvement due to intervention [117]. At the psycho-physiological level, brain functional imaging has located the neuro-chemical circuitry activated when participants expect they will receive, or believe they are receiving, a pain relieving intervention [116,118]. In fact, the changes in brain activity are similar to those occurring when genuine interventions are delivered [118,119,129]. As such, there is mounting evidence in support of a physiological basis for subjective constructs (*e.g. *expectancy and value) to produce powerful modulation of basic perceptual, motor and internal homeostatic processes [117]. However, it is proposed that the contributions of various neurotransmitters and neuropeptides involved in this placebo-induced, activity modulation might be disease- and symptom-specific [124]. Presently, no brain imaging studies have evaluated the placebo effect for foot pain interventions.

While it is desirable to minimise the magnitude of the placebo effect in clinical trials, it is possible that clinically meaningful benefits might be achieved by intentionally maximising the placebo effect in clinical practice [118]. More research is needed to determine if (and if so, how) this can be achieved. Until more clinically directive evidence is produced, clinicians should be aware that what the patient thinks, matters.

## Summary

In this review of foot pain, we have discussed its prevalence, aetiology and predictors, classification, measurement and impact. We have also described the complexities of foot pain as a sensory and emotional experience and how the psychosocial context can influence treatment response to produce a 'placebo effect'. It is hoped that this paper will provide a platform from which to advance the diagnosis and treatment of foot pain in clinical practice and its evaluation in clinical trials.

## Authors' contributions

FH searched the literature, retrieved articles and drafted the review. JB conceived the review, provided comments on content and made changes to the final document.
